# Stress responses in free ranging brown bears (*Ursus arctos*) in eastern Türkiye

**DOI:** 10.3389/fvets.2025.1639623

**Published:** 2025-10-03

**Authors:** Morteza Naderi, Rupert Palme, Kelly Yarnell, Emrah Çoban, Ayşegül Karaahmetoğlu Çoban, Josip Kusak, Çağan H. Şekercioğlu

**Affiliations:** ^1^Department of Biology, Faculty of Sciences, Sakarya University, Sakarya, Türkiye; ^2^Experimental Endocrinology, Department of Biological Sciences and Pathobiology, University of Veterinary Medicine, Vienna, Austria; ^3^School of Animal and Environmental Sciences, Nottingham Trent University, Nottingham, United Kingdom; ^4^KuzeyDoga Society, Kars, Türkiye; ^5^Division of Clinical Sciences, Department of Wild Animal Diseases, Faculty of Veterinary Medicine, Balıkesir University, Balıkesir, Türkiye; ^6^Veterinary Biology Unit, Faculty of Veterinary Medicine, University of Zagreb, Zagreb, Croatia; ^7^School of Biological Sciences, University of Utah, Salt Lake City, UT, United States; ^8^College of Sciences, Koc University, Istanbul, Türkiye

**Keywords:** brown bears, stress indicators, glucocorticoid metabolites, wildlife conservation, environmental impact

## Abstract

Brown bears (*Ursus arctos*) are a keystone species vital for maintaining ecological balance in northeastern Türkiye. However, increasing human activities—such as logging, agriculture, and recreation—pose significant threats to their conservation. It is therefore crucial to assess how these specific anthropogenic pressures influence bears’ physiological stress responses to inform effective conservation strategies. Our hypothesis that increased human activity would correlate with elevated stress markers in bears was tested by collecting blood serum samples from 50 free-ranging bears during live capture. Blood cortisol levels and fecal cortisol metabolites were measured to assess stress responses. We also employed camera trap surveys to quantify human activity levels across different habitat patches, calculating a Relative Abundance Index (RAI). Statistical analyses, including correlation and regression models, were used to assess relationships between cortisol measures, habitat features, and human presence. The study revealed an inverse correlation between bear mass and blood cortisol levels and a significant relationship between fecal cortisol metabolites and human presence, as quantified through camera trap data. These findings highlight the significant impact of human disturbances on bear stress physiology, the urgent need for effective conservation measures to minimize human-wildlife conflicts and support the long-term viability of bear populations in Türkiye. These findings highlight that fecal cortisol metabolites serve as reliable, non-invasive indicators of stress in free ranging brown bears, enabling large-scale monitoring to identify habitat disturbance hotspots, assess the effectiveness of protected areas, and inform targeted management actions to minimize human-wildlife conflicts and enhance habitat quality.

## 1 Introduction

Brown bears (*Ursus arctos*) are keystone predators and flagship species in many ecosystems, including northeastern Türkiye, where their presence indicates healthy forest and mountainous habitats (1). They play a vital ecological role by regulating prey populations and maintaining habitat diversity (2). Due to their keystone ecological role and cultural significance, brown bears are often a focal species in community-based conservation programs, encouraging local engagement in habitat protection and conflict mitigation. However, their populations face numerous threats in Türkiye, including habitat fragmentation, human-wildlife conflicts (3), and habitat degradation driven by logging, infrastructure development, and increased human presence (3, 4). These ongoing pressures threaten the long-term viability of Brown bears, underscoring the need for effective conservation strategies that incorporate biological monitoring, welfare, and stress assessment. Stressful situations, particularly those caused by human activities, can both directly and indirectly impact the long-term survival of wild species. This highlights the importance of ecological studies that focus on more vulnerable groups. Gaining insight into how these species respond and adapt to environmental shifts is essential for effective conservation efforts (5). Physiological indicators of animal welfare have been investigated among domestic animals (6, 7) animals in captivity (8), and rehabilitation centers, but this can be much more challenging while aiming for free-ranging animals (9). Among these physiological indicators, cortisol is the primary hormone used to assess stress responses in both domestic and wild animals due to its well-established role in the hypothalamic–pituitary–adrenal (HPA) axis.

In the context of welfare in wild, free-ranging animals, stress is not limited to direct threats or adverse events, but can also result from environmental factors such as habitat disturbance, species interactions, and human activity (9). They can include any species interaction, encounters with predators or rivals, and potentially beneficial interactions such as finding a mate, participating in social play, or engaging in vigorous physical activities. Anthropogenic sources, including human activity in wildlife habitats, can provide stressful conditions for wildlife, too (10).

In bears, blood glucocorticoids (serum GCs) have been measured as an indicator of stress [for an extensive review, see Babic et al. (11), Davenport et al. (12), Malcolm et al. (13)]. These hormones have also been utilized to assess stress responses related to capture techniques and to evaluate baseline levels specific to different bear species. Tryland et al. (14) stated that elevated cortisol levels indicated heightened stress in wild bear populations. Despite the broad use of blood glucocorticoids (GCs) to represent short-term stress status, studies also highlight high variability in cortisol levels depending on stimuli such as capture and handling, as well as factors like the time of day [referred to as “point” samples by (15)], therefore, measuring serum cortisol is not practical for assessing both short- and long-term stress in wildlife, making non-invasive techniques such as fecal cortisol metabolite analysis the preferred approach for studying stress in free-ranging animals (12, 16, 17). In conclusion, to evaluate the long-term stress state of free-ranging animals such as brown bears, it is essential to use reliable non-invasive methods that do not require animal handling or subject the animals to additional stressful procedures during capture and tranquilization. Such methodologies are critical for shaping conservation strategies in wildlife and free-ranging animals. These approaches enable frequent and cumulative physiological measurements with minimal, if any, disturbance to animals. Among them, measuring fecal cortisol and corticosterone metabolites (FCMs) has proven especially useful (see Palme (18) for a comprehensive review and evaluation of all previously published papers) (but for example see also: Hein et al. (19); Hunninck et al. (20); Babic et al. (11)).

FCM analyses in wild animals have been utilized to evaluate a range of stressors, including environmental, ecological, and anthropogenic factors. Natural environmental variables such as vegetation cover, prey availability, and weather conditions (e.g., temperature and precipitation) can significantly impact stress hormone levels, as they directly affect foraging success, reproduction, and overall habitat quality. Recent research on brown bears has demonstrated that physiological stress indicators such as blood cortisol and fecal cortisol metabolites (FCMs) reliably reflect responses to environmental and anthropogenic stressors (17). For example, Piñero et al. (17) conducted an ACTH challenge on unacclimated captive brown bears, illustrating the relationship between serum cortisol and FCM concentrations and highlighting the impact of human activity on stress hormone levels in natural bear populations. Hunninck et al. (20) reported that environmental factors like vegetation cover and quality can significantly influence FCM levels in wildlife, reflecting annual variations in food resources and habitat conditions impacting stress responses in species like impala (*Aepyceros melampus*) (20). Similarly, in red deer (*Cervus elaphus*) and chamois (*Rupicapra rupicapra*), precipitation and temperature were found to significantly influence FCM concentrations, indicating that seasonal environmental changes can modulate stress responses (21). FCM studies have also been carried out on free-living mountain hares (*Lepus timidus*) (22), capercaillies (*Tetrao urogallus*) (23), red deer (*Cervus elaphus*) and roe deer (*Capreolus capreolus*) (24), Alpine chamois (*Rupicapra rupicapra*) (25), Geoffroy’s spider monkeys (*Ateles geoffroyi*) (26), and Mexican gray wolves (*Canis lupus*) (27).

In some cases, human-related factors like habitat fragmentation, noise, and direct human presence have been shown to induce higher stress levels than natural ecological pressures, such as predator presence (24). These studies suggest that the impact of human activities can overshadow or interact with natural stressors, influencing wildlife behavior and physiology in complex ways. However, the length of exposure to human-induced disturbances can be significant in influencing the magnitude and duration of stress responses in wildlife. For example, a study on chipmunks (*Tamias striatus*) in urban habitats showed that they experience less stressful conditions than their nature-dwelling counterparts (28). Rangel-Negrin et al. (29) found a positive relationship between FCMs and environmental disturbances, with the lowest amounts of FCMs found among individuals in protected areas where the anthropogenic disturbances were the lowest (29). The authors also reported that habitat fragmentation can be a long-term stressor for spider monkeys. Moreover, anthropogenic activities can also exert a context-dependent effect on animals’ stress physiology (22, 30). However, studies on large carnivores’ welfare have focused less on the impact of habitat fragmentation, habitat patch size, seasonality, and human activity, but have focused on captive animals (4, 29–32).

Our study provided an ideal opportunity to explore physiological stress indices in free-ranging brown bears across a human gradient, which can then be compared to samples from rehabilitation centers in future studies, to find verified stress indices and inducing factors. We explored the relationship between serum GCs and FCMs and whether higher human activity, such as logging, would impact animals’ stress levels as measured via FCMs. This study aims to test the hypothesis that increased human activity correlates with elevated stress indicators in free-ranging brown bears, and to evaluate whether fecal cortisol metabolites serve as reliable non-invasive biomarkers of long-term stress in natural habitats. This research should advance our understanding of the spatial and human-induced factors that affect the stress physiology of free-ranging brown bears, and the findings can be applied to enhance their welfare.

## 2 Methods

### 2.1 Study area

**Figure 1:**
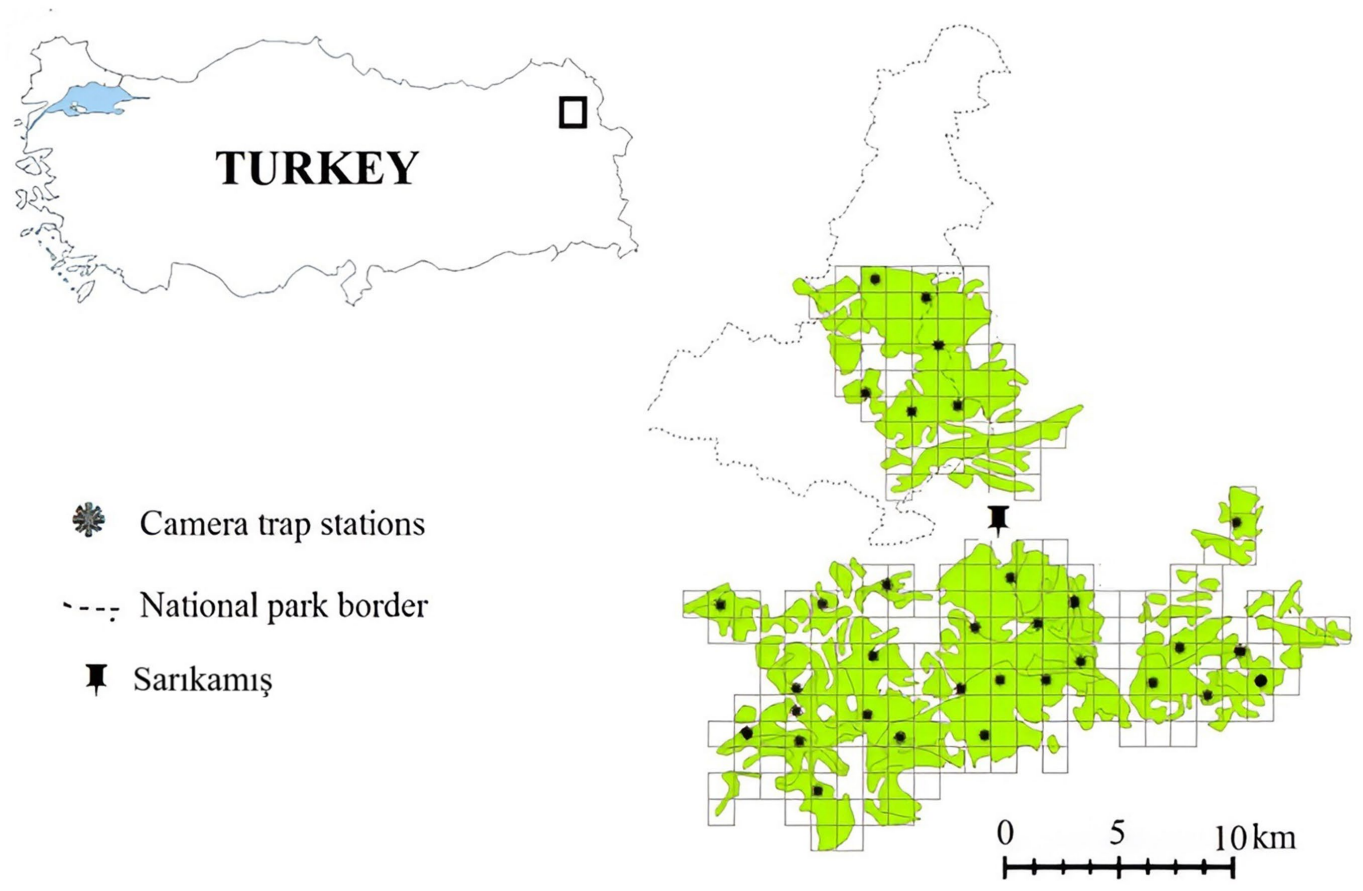
Study area and the location of the camera trapping stations. The black points show the camera traps.

Sarıkamış forests- Allahuekber Mountains National Park in north-eastern Türkiye is home to Brown bears (*Ursus arctos*), Gray wolves (*Canis lupus*), and Caucasian lynxes (*Lynx lynx dinniki*) (33, 34) with a relatively high degree of large carnivore-related human-wildlife conflict (34). Villagers inhabiting areas around the forests and organizations as forestry management, routinely use these fragmented Scotch pine forest patches with a total area of 338.5 km^2^ for logging (1) (Figure 1). Livestock herding, recreation (picnicking), and wild herb and mushroom collection create a high level of human activity, except during the harsh winter season (35). The ambient temperature usually does not fall below 20 °C during the warm season (May–October) but reaches levels lower than −10 °C during the cold months (November–April). Mixed beach-fir forest, mostly *Fagus sylvatica*, *Abies alba*, *Picea abies*, *Quercus* sp., *Castanea sativa*, and *Pinus* sp., constitute the forest community type in the study area (36). Utilizing the Relative Abundance Index (RAI) from camera trap surveys, we quantified human activity intensity within each patch, correlating these findings with physiological stress indicators in bears. The Relative Abundance Index (RAI) is a commonly used metric in camera trap studies that estimates the activity or detection rate of animals or humans within a given area, standardized by sampling effort. It is calculated by dividing the number of detection events at a station by the total trap days and multiplying by 100, providing detections per 100 trap days as an indicator of relative activity levels. Villagers around these forests engage in activities like logging, livestock herding, recreation, and wild herb collection, barring the severe winter months. An open garbage dump is located across the bear’s distribution at the study area and affects behavioral patterns in bears, including seasonal migration habits (35). There is considerable human-wildlife conflict at the garbage dumps, as bears are often drawn closer to human settlements due to the readily available food resources, leading to increased interactions, potential conflicts, and sometimes road mortalities. The Sarıkamış area provides a unique case for substantially advancing brown bear welfare and stress indices.

### 2.2 Camera trapping

A large carnivore study and conservation management project has been performed in the study area during the past 13 years, led by the KuzeyDoğa Society (KD). We used data from camera trapping surveys from 2018 to 2023 to quantify the human activity in the area. During this period, 28–42 cameras (Reconyx Ultrafire XR6 and Reconyx PC900) were deployed systematically throughout the study area, divided into two square km grids, ensuring equal distribution of the cameras in each habitat. A total of 75,000 trap days have been done to collect data during the 6 years. Camera stations were set along roads and intersections of minor forest roads and wildlife trails without using any bait or attractant (37). We installed all the cameras 2 m high in the trees. Cameras were checked regularly every month. Based on Kays et al. (38), all cameras were set to take a series of five photos per trigger without any delay, with medium sensitivity and a 30-s sensor break between series (38). During this study, cameras were routinely shifted based on a uniform random pattern (39) to different study grids (generated in ArcGIS software ver. 10.3.1, ESRI, Redlands, CA) to cover most grid cells (4). To assess the level of human activity within the study area, we utilized the Relative Abundance Index (RAI). This approach allows for comparison across different locations and periods, accounting for variations in sampling effort. In our study, the RAI served as a proxy for human activity intensity because a higher number of detections at a station indicated increased presence or activity of humans in that area. This standardized measure was then used to explore relationships between human disturbance and stress hormone levels in brown bears (4).

### 2.3 Sample collection

This research forms a segment of an ongoing conservation and population biology initiative focused on the large carnivores of northeastern Türkiye, including brown bears, wolves, and lynxes, spearheaded by the KuzeyDoğa Society since 2006 (40). Bears were captured using Aldrich snares in a “European cubby” (41). A GSM-equipped camera trap was also installed to send instant photos of the possible capture, and the intervention time was then reduced to around 20 min for handling the captured bear. We also used GSM-equipped alarms to send signals on any triggering of traps. In this study, we used a tranquilization protocol involving a combination of Zoletil 100 and Domitor. The dosage was 2.5 mg/kg of Zoletil 100 and 0.05 mg/kg of Domitor. This combination was prepared such that 0.25 mL of the mixed solution was used for each 10 kg of body mass (42). Target animals were then equipped with Vectronic Aerospace GPS-GSM/Iridium collars (Vectronic Aerospace GmbH, Berlin, Germany) with two-axis activity sensors, which continuously recorded the acceleration and stored the values within a range of 0–255 in five-minute intervals. Standard capture forms were filled out for each captured animal, and all necessary data for this study, such as sex, age, and mass, have been recorded. Blood samples were taken from the femoral vein (*Vena femoralis*) and stored in the fieldwork station’s cool box for less than 1 h and centrifuged at 3,000 rpm for 5 min. Separated plasma samples were stored in a −20 °C freezer for laboratory analysis.

In all cases, captured bears’ fresh scat samples were found around the cubby and immediately transferred to the −20 °C freezer. Collected scats from the mentioned cubbies were very fresh, less than 30 min after defecation. The other scats across the habitats were also selected from the fresh ones that had been defecated during the previous night. In some places, such as the garbage dump, we also collected less than 10 min old scats. As a unique study on free-ranging brown bears across the world, we collect the largest sample size of bears’ blood (*n* = 50) and 23 scat samples known to belong to the same individuals from which the blood samples were taken. Those scats were categorized based on the animal’s sex since they were collected along with the animal’s live capture while monitored by the real-time cameras installed at the cubbies. For the rest of the scat samples (*n* = 28) the sex could not be detected as they were collected in different parts of the habitat. We also followed collared bears to exact locations, including dens, daily beds, and resting areas, allowing further fresh scats to be collected. Since the study area has an open garbage dump, some collared bears spend their time foraging in the garbage dump for food. Therefore, by visiting the garbage dump, we could spot the collared bears and collect fecal samples from this area while also determining the sex of the collected scats. This allowed us to explore the impact of anthropogenically driven feeding areas on animal stress status as the feeding area was often shared with dogs that vocalized often. To control for potential seasonal effects, samples were collected throughout only the warm season (May to August), and sampling was evenly distributed across different months of this season; therefore, the seasonal effects have not been considered in this study. All procedures aimed to minimize temporal variability, but inherent seasonal influences on hormone levels were acknowledged as a limitation. Table 1 indicates the number of samples with more details.

**Table 1:** Collected samples based on type and sex.

Sample type	Females	Males	Unknown	Total
Scat	11	12	28	51
Blood	11	39	0	50

### 2.4 Steroid analyses

For FCMs, we weighed 0.5 g of each wet scat and then added 5 mL of 80% methanol to the sample. Each falcon tube was vortexed by hand for 2 min, and all samples were centrifuged for 15 min at 2,500 g. Finally, 0.5 mL of supernatant was transferred to new tubes and dried in an oven at 40–50° C for 24 h (43). For serum samples, we took 0.5 mL of serum and added 5 mL of diethyl ether and kept it in the 15 mL falcon tubes, shook it by hand several times, and centrifuged it (15 min at 2,500 g). Falcon tubes were frozen for 2–3 h. Then, gently, we transferred the ether phase to new falcon tubes using micropipettes. Afterwards, the ether phase was dried and redissolved in an assay buffer. FCMs were determined with a cortisol enzyme immunoassay (for details, see Palme and Moestl (44)), which has been successfully validated for brown bears (45). The same cortisol assay was used for the serum extracts.

### 2.5 Statistical analyses

First, normality of the datasets (serum cortisol, fecal cortisol metabolites) was assessed using the Shapiro–Wilk test for all samples and relevant subgroups (e.g., by sex, mass). For normally distributed variables, parametric tests such as Pearson correlation and *t*-test were employed; otherwise, non-parametric alternatives like Spearman correlation and Mann–Whitney U test were used. To determine the relationship between FCMs and human activity, the Spearman’s rank correlation coefficient was calculated. The correlation between blood cortisol and fecal cortisol metabolite concentrations within the same individuals was also assessed using Spearman’s correlation. Differences in cortisol levels between sexes were examined using the Mann–Whitney U test, due to small sample sizes and non-normal distribution of subgroup data.

Comparisons of FCM levels among different habitat patches were conducted with the Mann–Whitney U test, as the data did not meet parametric assumptions. All significance levels were set at *p* < 0.05. Assumptions of each test were checked using residual plots and appropriate diagnostics. Analyses were performed using R, with mentioned packages like ggplot2 and lme4 for statistical modeling and data visualization, and significance levels were set at *p* < 0.05. Data normality and assumptions for each analysis were checked and addressed accordingly to ensure the reliability of the results. Additionally, we considered the potential influence of covariates, such as age and mass, in the analyses to enhance the accuracy of our interpretations.

### 2.6 Ethical declarations

All animal procedures followed the Kafkas University local ethical committee for animal experimentation (KAÜ HADYEK) guidelines under KAÜ HADYEK/ 2018-050 and KAÜ-HADYEK/ 2021-083 permission number. Capturing bears was conducted under strict ethical guidelines approved by Turkey’s Department of Nature Conservation and the Ministry of Agriculture and Forestry (Permit No. 72784983-488.04-114100 and E-21264211-288.04-1602322). The procedure was justified by the necessity to obtain biological samples (blood and feces) from individual bears for stress assessment, which cannot be reliably achieved through non-invasive means alone in this context. During immobilization, bears were monitored continuously, and vital parameters were checked regularly. Bears were kept under supervision until full recovery from anesthesia.

## 3 Results

**Figure 2:**
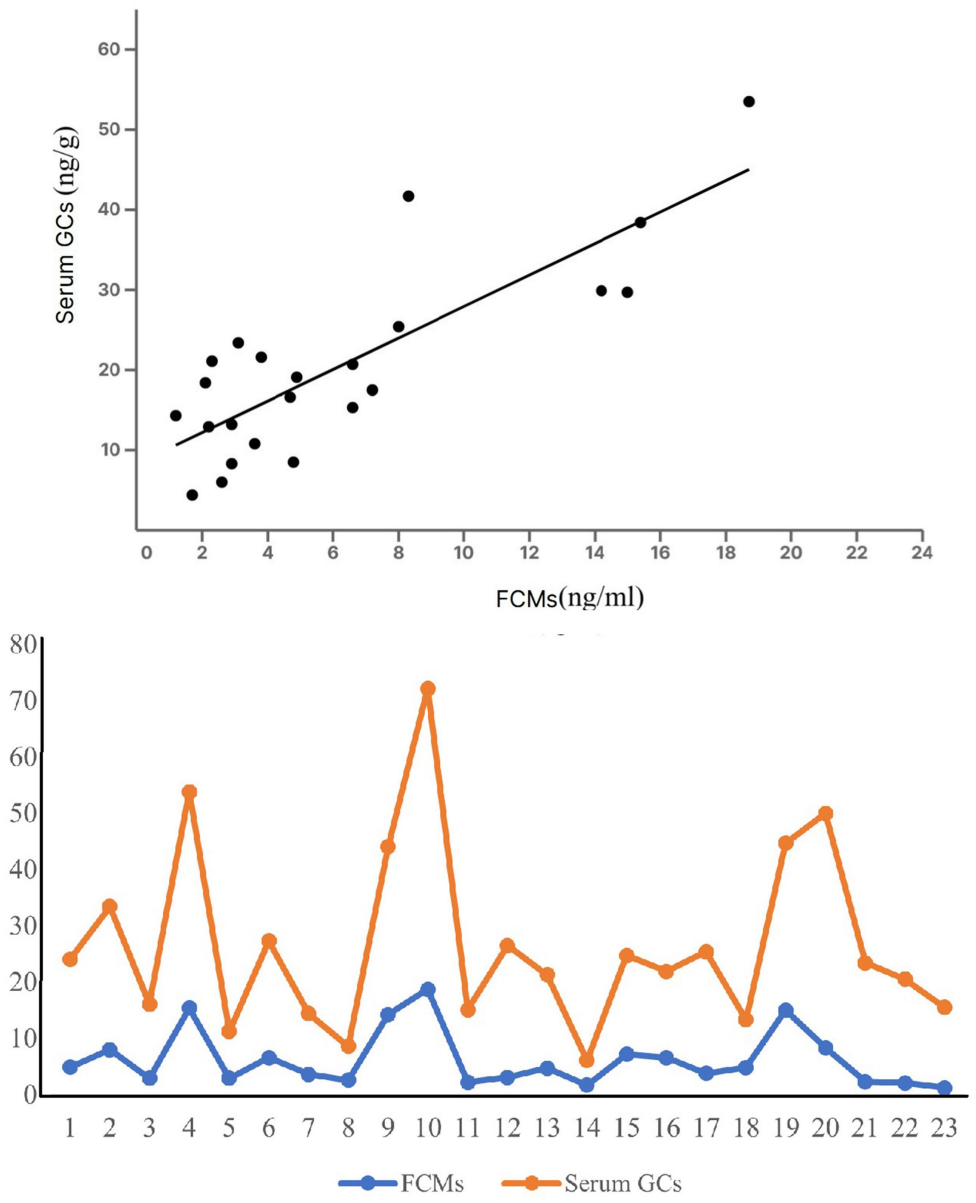
A linear relationship between FCM and blood cortisol concentration in the same individuals.

Blood cortisol levels ranged from 2.3 to 58.5 (median: 17.9) ng/ml, and FCM concentrations from 1.2 to 16.1 (median: 4.5) ng/g. These ranges come from the whole data, including blood and scat samples taken from the same individuals. There was a relationship between levels of blood cortisol and its metabolites in scat samples of the same individuals (*r* = 0.61, *p* < 0.05) (Figure 2). To verify the assumption of normality for our dataset, we conducted the Shapiro–Wilk test across variables. The pooled data, not classified by age or sex, demonstrated a *p*-value of 0.22, indicating a normal distribution. We investigated the correlation between animal mass and blood cortisol levels using Pearson’s correlation coefficient. Our analysis revealed a significant inverse correlation (*r* = −0.41, *p* = 0.04, *n* = 50). This suggests heavier animals tended to have lower cortisol concentrations.

**Figure 3:**
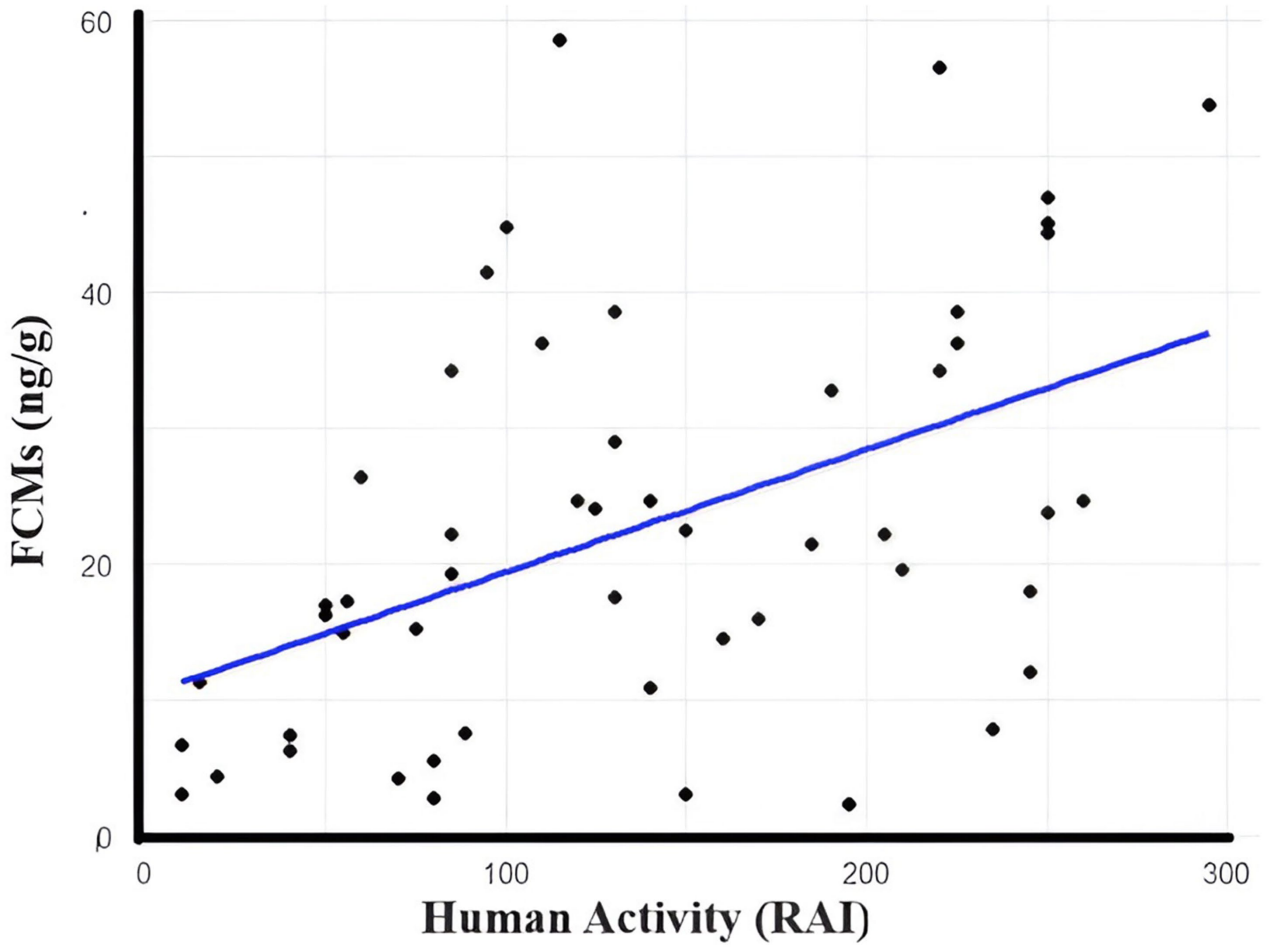
Human activity (RAI) and stress levels of brown bears. This graph indicates that the animals suffer from more stressful conditions in areas with higher human activity.

We conducted a Mann–Whitney U test to evaluate the differences in blood cortisol levels between male and female bears. The analysis did not reveal a significant difference in cortisol levels between male and female bears. Due to the presence of tied values—where multiple observations share the same measurement—in our data, the Mann–Whitney U test was conducted with a correction for ties, which adjusts the test statistic accordingly. The results revealed a U statistic of 159 and a *Z* value of −0.9674. The analysis failed to reject the null hypothesis, as the *p*-value was 0.3 (*U* = 159, *Z* = −0.9674, *p* = 0.3). This indicates no statistically significant difference in blood cortisol levels between the sexes. The computed standardized effect size was small (0.14), and the observed common language effect size was 0.4, suggesting a low probability (40%) that a randomly selected cortisol level from a male bear would exceed that from a female bear. In this study, we found a good correlation between measured FCMs and human activity (*r* = 0.71, *p* < 0.01; Figure 3), which was estimated from the camera trapping project (calculated as RAI). We found lower mean FCM levels in samples collected from open garbage dumps than in the habitat patches. However, differences were not statistically significant (Z equals 1.34). There was no significant relationship between FCMs and sex/age (*p* > 0.05).

## 4 Discussion

Despite recent advances in understanding stress physiology in free-ranging mammals, there remains a significant gap in knowledge regarding the welfare and stress conditions of free-roaming animals such as large carnivores that inhabit large, unbounded areas and encounter a wide range of environmental challenges (9). Specifically, for large carnivores such as brown bears, data on long-term stress indicators are limited, particularly in their natural habitats where multiple anthropogenic and ecological factors intersect. In this study, we explored blood serum cortisol and FCM concentrations utilizing the largest sample size from free-ranging brown bears at their natural habitats, since most similar studies have been carried out on captive brown bears (17). Both measures were correlated, and we found an inverse correlation between bear mass and blood cortisol levels and a significant relationship between FCMs and human presence, as quantified through camera trap data.

We believe that our findings can contribute to advancing research of this type, particularly focusing on large carnivores like brown bears. Our samples were highly valuable and unique because we were able to collect both blood and scat samples from the same individuals in their natural habitat. Therefore, we were able to evaluate the relationship between blood cortisol and FCM concentrations, which has also been done in a previous study (17). In this research, we found that FCMs can be a suitable index for free-ranging bears’ welfare and stress status across their natural habitats, because their levels are not influenced by capture stress. Blood cortisol concentrations can be highly variable about the environmental conditions (15) and needs animal live capture and immobilization, which can be highly stressful and expensive (17).

Previous studies suggest that body condition can influence an animal’s stress response (46, 47). Lower cortisol levels in heavier bears suggest that larger, potentially more dominant or better-conditioned bears might experience lower physiological stress, possibly due to better access to resources or superior territory. Larger bears may also be more successful in securing high-quality habitats, which could buffer them from certain stressors such as human disturbance. Conversely, smaller or subordinate individuals might experience higher stress levels due to increased competition or limited access to resources, which could be reflected in increased cortisol. However, this relationship warrants caution; higher body mass may also be associated with other factors like reproductive status or age, which we did not fully control for. Therefore, while a larger size might suggest better access to resources or higher social standing, it’s important to recognize that the relationship between size and stress is complex and may involve various ecological and behavioral factors. Future studies incorporating detailed habitat use and social hierarchy data could provide deeper insights into how size and territoriality modulate stress responses in brown bears.

The lack of significant relationships between FCM or blood cortisol levels with age or sex highlights the complexity of stress responses in wildlife, which has also been confirmed by previous studies. For example, Pinero et al. (17) also reported that there was no relationship between sex and both blood cortisol and FCM concentrations. However, this does not mean that the situation can be the same among other large mammals. For example, an opposite finding has been reported for Coyotes (*Canis latrans*) (48). These results suggest that factors other than age and sex, such as environmental conditions and individual health status, may play more crucial roles in determining stress hormone levels in brown bears (49, 50). Human activity emerged as a critical factor influencing bear stress levels. The significant relationship between human presence (as measured by RAI) and higher FCM concentrations indicates that bears in areas with higher human activity experienced increased stress. This finding is consistent with other studies documenting the impact of anthropogenic disturbances on wildlife stress physiology (24, 51). Human activities such as logging, livestock herding, and recreational activities can create disturbances that elevate stress hormones in wildlife (22).

Other studies indicated a relationship between FCMs and long-term stressful conditions, such as anthropogenic activities and stress conditions inferred from FCMs. For instance, Asiatic black bears (*Ursus thibetanus*) had higher levels of FCMs outside nature reserves with higher human activities (13). Nevertheless, Babic et al. (11) indicated that FCMs may not accurately reflect chronic or long-term stress or the impact of habitat conditions on bear welfare due to various factors such as adaptations, reproduction status, hibernation, etc.

While not statistically significant, comparing samples from garbage dumps and natural habitats provided valuable insights. The lower mean FGM levels in garbage dump samples could be attributed to the availability of accessible food resources, reducing the need for bears to engage in energy-intensive foraging activities. However, the lack of statistical significance suggests that other factors, such as the presence of dogs and humans, may offset the potential stress-relieving effects of accessible food sources (35, 52). Bears exposed to higher levels of human traffic exhibited increased cortisol concentrations, indicating heightened physiological stress (52). Roads and human presence can fragment habitats and create barriers to movement, leading to increased stress and decreased habitat quality for wildlife (3, 4). This study demonstrates that fecal cortisol metabolites are reliable, non-invasive indicators of long-term stress in free-ranging brown bears, closely correlating with serum cortisol levels. Our findings highlight the impact of human activities on bear physiology, emphasizing the importance of minimizing disturbances and habitat fragmentation. Incorporating stress biomarkers into conservation strategies can enhance monitoring efforts, identify stress hotspots, and support long-term population sustainability. Future research should expand spatial and seasonal sampling, include behavioral data, and examine cumulative effects of multiple stressors, aiding the development of adaptive management plans that prioritize both species welfare and ecosystem resilience.

### 4.1 Study limitations

This approach provided valuable insights into how these stress markers relate under real-world conditions. However, several limitations must be acknowledged. First, the uncontrolled conditions inherent to wild environments—such as variable diets, fluctuating weather patterns, and differences in individual activity levels—may influence hormone levels independently of human disturbance or other stressors. Additionally, physiological measures like cortisol are subject to natural circadian and seasonal variations (18), which can complicate interpretation when sampling occurs at different times or seasons without precise control. Furthermore, factors such as animal age, reproductive status, health condition, and prior exposure to stressors were not fully controlled or standardized in our sampling process. These variables can potentially influence hormone levels and confound the relationship between observed stress responses and environmental factors. Finally, as with all field-based studies, the inability to manipulate external variables limits the extent to which causality can be established. Despite these limitations, we believe our findings provide meaningful contributions toward understanding the stress physiology of large carnivores in their natural habitats and can serve as a foundation for future, more controlled research. We suggest that subsequent studies incorporate repeated measures across seasons and habitats, alongside controlled assessments of diet and activity budgets, to better isolate the effects of specific stressors. Moreover, integrating GPS movement data and behavioral observations could help contextualize hormonal fluctuations with behavioral responses, providing a more comprehensive understanding of animal welfare under varying environmental conditions.

## Data Availability

The raw data supporting the conclusions of this article will be made available by the authors, without undue reservation.
